# Complex systems perspective in assessing risks in artificial intelligence

**DOI:** 10.1098/rsta.2024.0109

**Published:** 2024-11-13

**Authors:** Daniel Kondor, Valerie Hafez, Sudhang Shankar, Rania Wazir, Fariba Karimi

**Affiliations:** ^1^ Complexity Science Hub, Vienna, Austria; ^2^ Independent researcher/Women in AI Austria, Vienna, Austria; ^3^ Graz University of Technology, Graz, Austria; ^4^ leiwand.ai, Vienna, Austria

**Keywords:** artificial intelligence, risks, complex systems

## Abstract

In this article, we identify challenges in the complex interaction between artificial intelligence (AI) systems and society. We argue that AI systems need to be studied in their socio-political context to be able to better appreciate a diverse set of potential outcomes that emerge from long-term feedback between technological development, inequalities and collective decision-making processes. This means that assessing the risks from the deployment of any specific technology presents unique challenges. We propose that risk assessments concerning AI systems should incorporate a complex systems perspective, with adequate models that can represent short- and long-term effects and feedback, along with an emphasis on increasing public engagement and participation in the process.

This article is part of the theme issue ‘Co-creating the future: participatory cities and digital governance’.

## 1. Introduction

Recent advances in artificial intelligence (AI) systems have intensified the debate about potential risks posed by them and the potential for regulation that could mitigate them [1–6]. These advances echo earlier concerns that have been voiced for the deployment of AI systems in many domains [7–13]. Such concerns have recently prompted legislators to take action, with the European Union’s Digital Services Act (DSA) [14] and, more recently, the AI Act [15,16] being among the most comprehensive efforts for regulation with explicit aims of identifying and mitigating risks.

Despite such interest, there are several unresolved questions about risk assessments, and it is yet unclear what effects legislative efforts by the EU or other governments will have. What is deemed risky about AI can often differ vastly between specific groups of thought. Within the community of practitioners, there is even basic disagreement about whether the group of methods and technologies commonly called AI is *itself* inherently risky or whether the *application* of these methods and technologies for specific purposes is risky. A growing body of research has provided examples of the latter, including biases and harmful outcomes arising in many use cases [3,7–10,13]. These are contrasted by discussions on more fundamental, emergent risks that could involve a future transformative event as a consequence of runaway technological growth [1,12,17–19]. The AI Act also features this dichotomy: while it mentions *systemic* risks that could arise if a certain data processing threshold has been reached (corresponding to $10^{25}$ floating point operations per second; Art. 51 AI Act), its main focus is on risks arising within specific use cases. This includes transparency obligations imposed on certain uses and contexts and the specific enumeration of risks to health, safety and fundamental rights as areas of explicit concern (Art. 9(2), also Annex I and III AI Act [16]).

The debate around risk in relation to AI is further complicated as risk can be conceptualized differently and, thus, needs to be placed in relation to harms. Even with a standardized definition of risk such as the one provided in the AI Act, open questions remain about what is put at risk through an AI system. Considering more specific risks to health and safety, it is important to be clear whose health or safety should be considered. Given the potential wide-ranging and indirect effects of certain technologies [8,12,20], a narrow definition (e.g. people who use or are directly affected by an AI system) could miss important indirect outcomes. Additional questions arise when considering systemic interactions, e.g. whether safety includes the safety of critical infrastructure or whether mitigating ‘risks to fundamental rights’ requires active measures for providing or maintaining such rights, or only prevention of encroachment of such rights by governments or private actors under specific circumstances (cf. discussions on ‘positive’ or ‘negative rights’ in political science [21,22]). More generally, risks can depend on complex interactions of technologies with the social, political and economic context in which they are deployed [12,23–26], and thus, a reliable assessment of a full catalogue of risks might not be feasible.

In addition to the ontological issue of what constitutes a risk to what or whom, there are additional issues of how risk is measured and interventions to mitigate risks or harms are implemented. Given the wide-ranging potential effects and uncertainties, limitations and weaknesses in risk assessments need to be seriously considered when they form the basis of policies and legal actions. Both research and policy-making should consider ways to increase public participation, opening up decision-making processes and allowing more input from people whose life is affected by the deployment of new technologies [6].

In this article, we argue that to better understand the potential effects and risks of AI, we need a complex systems perspective that can incorporate feedback and long-term uncertainties [27–29], along with a critical evaluation of how research and policy-making are integrated in society [30]. We believe that such an approach can effectively connect questions about specific and systemic risks by viewing the deployment of AI systems and regulatory or social responses to them in the more general economic, social and political environment in which they operate. Importantly, instead of a dichotomy between specific and systemic risks where the latter concern a presumed ‘transformative’ future event [1,17,18,31], we consider systemic risks as long-term consequences of technological development and deployment [8,19,30,32] where specific problematic uses of AI systems may add up to a future that is misaligned with the goals and values of our society [33]. Thus, we argue that the debate about the risks of AI technologies also needs to better integrate perspectives about specific, individual- or community-level harms and how these contribute to long-term changes in the wider social context [34–38]. Such a view will also acknowledge that outcomes will depend not only on policy related to AI but also on emergent processes that are influenced by the wider social context, including aspects unrelated to AI [12,34,35,37,38]. In the following, we aim to identify key issues and themes related to the above and propose both research directions and policy interventions based on them.

This article is structured as follows: we begin by examining risk assessment practices and highlighting potential issues when applying them to AI systems in §2. We continue with a discussion of two areas that we believe are especially relevant when thinking about long-term risks: the interaction of AI systems with inequalities (§3) and political participation (§4). In §5, we then present a case study discussing the deployment of a large-scale automated system with an explicit goal of preventing inequalities and identifying key issues that relate to the previously discussed concepts. Finally, in §6, we present several directions for improving risk assessments, including an illustrative example of a simulation model about the short- and long-term impact of algorithms on the visibility of minorities, a discussion on increasing public participation via competency groups and how these come together in a complex systems approach to risk assessments. We end with some concluding remarks in §7.

## 2. Risk assessments in artificial intelligence

In this article, we use a broad definition of AI that includes any ‘complex information processing system’ [39] capable of being used in decision-making processes on large scales. While recent developments with *generative* AI systems have brought the debate about the risks of AI to wider public attention [3–5], many of the questions and issues raised are neither entirely novel nor unique to any specific AI technology. Similar problems have already been discussed in relation to ranking or decision-making solutions that involve the aggregation of large amounts of data or information [13,36,38,40,41]. However, recent advances in computational capabilities and in the amount of data collected mean that such solutions will be possible to deploy in a wider range of domains, with a higher possible impact on societies. At the same time, AI systems based on deep learning present additional challenges, primarily stemming from their architecture being opaque to many established forms of reasoning and analysis [4,24,25,42,43].

### (a) Defining risk

**Figure 1.**
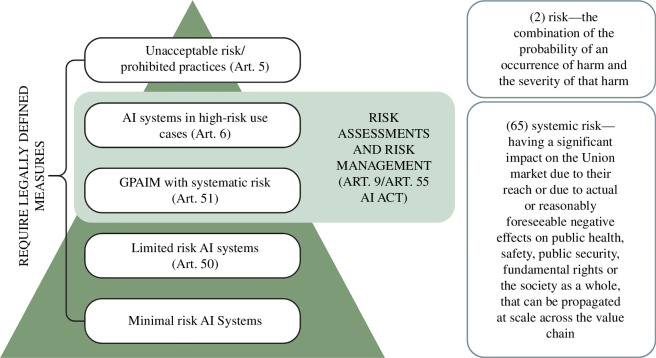
Overview of risk assessment requirements in the AI Act. Risks are organized based on their severity. Definitions of risk and systemic risk are laid out in Article 3(2) and Article 3(65).

With the passing of the EU’s AI Act [15,16], there is an increased interest in conceptualizing, characterizing and quantifying potential risks from the use of AI technologies or, more generally, the widespread use of automated systems for decision-making [3,24,44]. For high-risk AI systems as well as general-purpose AI models (GPAIM) with systemic risk, the AI Act introduces requirements for identifying, assessing and managing risks (see figure 1). The AI Act defines ‘risks’ as well as ‘systemic risks’ alongside prohibited and high-risk use cases and specifies in Art. 9 that the risk management should address risks posed to health, safety or fundamental rights (which, in Art. 1, is defined to encompass democracy, the rule of law and environmental protection). In the AI Act, systemic risks remain underdefined, and it is important to note that systemic risks in the DSA [14] appear to differ from systemic risks in the AI Act (although it is possible that they, as a course of practice, will begin to overlap; however, this is not a legal requirement). At the same time, definitions of specific risks encompass processes that happen on different timescales and contexts. For example, risks to health can be short- or medium-term and relate to outcomes affecting a specific person; on the other hand, risk to democracy could encompass processes that happen over decades and affect the potential for political participation for millions of citizens in subtle ways—something that could be conceptualized as systemic risk as well.

We, thus, believe that the above questions about defining risk will make it challenging to operationalize risks in a way that can guide policy, and thus, further work is needed on exploring the connections between specific and systemic risks. In our thinking, the latter encompasses risks emerging within the contexts created and affected by AI systems. Thus, they may emerge from the interaction of several factors, which, individually, may be considered negligible within established frameworks or in isolation [23,27,29,33,44,45]. In other words, risks considered in the AI Act to be high or systemic can arise via complex interactions of AI systems with the social context in which they are deployed [12,24,25]. We note that risk assessment practices will need better ways to explicitly take into account complex feedback, and to do so, they need to allow wider definitions of risks (especially ‘systemic’ risks) that present challenges when connecting scientific and legal definitions.

### (b) Assessing risk

Beyond issues around defining risks, further questions arise when risks need to be assessed based on any definition. First, risk assessment is widely considered to be an expert activity [30]. When risks are defined and then assessed, this process is embedded within institutional and societal power structures and draws on the epistemic frameworks of those immediately involved in the risk assessment [30,45] (see also §4), who in turn draw on wider or established conceptualizations and practices (as shown, e.g. by [3,44] discussed below). To make a crude divide, expertise (of a certain kind) about ‘AI risks’ is usually given pre-eminence to the embodied experience of harms caused through AI systems. Such issues have been pointed out in other technoscientific controversies [46], e.g. when the epistemic practices of policymakers and the agricultural industry conflict with the experience-oriented epistemic practices of beekeepers in the controversy around neonicotinoids [45,47], or in the case of AI-driven automation, where questions about efficiency gains clash with concerns about job loss and its spillover effects to communities disproportionally affected by it [37]. What counts as evidence for or against certain risks can, therefore, not be considered the outcome of a value-neutral process but dependent on the positionality of the actors proposing the evidence [28].

Second, risk assessment is an activity that requires assembling known issues as well as unknown factors [30]. Framed within the established paradigm of known-knowns, known-unknowns, unknown-knowns and unknown-unknowns, risk assessment relies primarily on known-knowns and known-unknowns (such as when classifying predictions as false positives or true negatives), perhaps may even help identify unknown-knowns but can say little about unknown-unknowns [48]. Paradoxically, what is used as evidence for certain risks (or their absence) may count as either knowledge or non-knowledge for certain actors within their own epistemic frameworks [45,47,49]. For instance, the absence of evidence for human extinction caused by AI may be translated into either the claim that we do not have evidence for this scenario to be a realistic risk, or it could be interpreted as a limitation of our risk assessment methodologies [1]. Risk assessments can be severely limited in complex systems where estimations of future outcomes are intractable and strong emergence can lead to unexpected behaviour [27,29], while any intervention based on risk assessment has to be considered part of the system itself [28]. Building on previous experience when considering new phenomena can lead to over- or underestimation of certain risks, leading to difficulty in finding effective risk mitigation measures as has been demonstrated recently for the case of online misinformation [50]. For such complex interactions, ensuring the diversity of experiences within the risk assessment can help address a more robust set of risks. This also points to the need to work with frameworks that explicitly acknowledge the unknown and the unknowable and make these uncertainties explicit to other actors, especially given the diverse possible ways in which AI systems could interact with their socio-economic context [12].

Third, risk assessment entails not only the identification of risks but also their classification and valuation. That is, risks need to be sorted out according to particular characteristics, e.g. the likelihood of their occurrence and the potential damage caused in the case of their occurrence (which is indeed both the framework proposed by the AI Act and the most widespread definition of risk), using specific thresholds to aid classification. Risk assessment itself can, therefore, be seen as a classificatory process [51], the effects of which spread beyond the initial classification of an AI system as risky to the actual risks posed by an AI system. Risk assessments should, therefore, be reflexive of their own riskiness—that is, the potential damage caused by risk assessments being incorrect. This is especially important given the possibility that such risks might be borne disproportionally by already disadvantaged groups whose participation in risk assessments is currently limited [8,13,37,38].

Risk assessments in connection with AI systems leave many of these questions unresolved. While established practices in other domains can serve as models, their applicability can be limited due to the complex interactions of AI systems and society [12]. Recent works still often focus only on specific, short-term risks, such as the cybersecurity aspects analysed by Panjakshan *et al*. [3]. While fruitful as a starting point for methodically approaching risk assessments, the risk matrices of such assessments often rely on previously identified risks and have difficulty expressing interactions between risks. Future risk assessments could build on such results by integrating them with models and frameworks that consider long-term feedback and interactions of different kinds of risks. Such an attempt to introduce more nuance and thereby improve risk assessment practices has been made by Novelli *et al*. [44]. These authors take the risk assessment model by the Intergovernmental Panel on Climate Change, developed further by Simpson *et al*. [52], to offer a layered model of risk assessment which, at the first layer, seeks to understand interactions between determinants of a risk (that is, hazards, vulnerabilities, exposures and responses). At the second layer, the risk model evaluates interactions of drivers between risk determinants as well as within risk determinants (the latter being bidirectional, unidirectional or aggregate). Both the first and the second layers refer to one risk, e.g. the risk of inequality. At the third layer, the risk model seeks to understand interactions between risks—e.g. risks to equality, privacy and environmental protection—which may be aggregate, compound (unidirectional or bidirectional) or cascading. The approach by these authors offers significantly more consideration toward complex interactions, but the risk categories it is based on again derive from predetermined risks—in this case, the AI Act.

These approaches show only a limited degree of reflection on the epistemic practices associated with risk assessments. Instead, these risk assessment methodologies focus on the risks (as out-there, independent from their observation) and not on the model for assessing the risks, who can participate in risk modelling and how uncertainties regarding the model for risk assessments are handled. It is in precisely in this area where improvements can be made by facilitating participation in the determination and evaluation of risks.

## 3. Artificial intelligence systems and inequality

A key issue that has been raised in many recent works is the potential for AI systems to contribute to growing social and economic inequalities [13,26,38]. At the same time, growing inequality has been identified as a key social issue by many authors [53–56]. We note that technological, economical and political changes are often interlinked. However, the full effects of technological change were rarely planned for or even realized in advance, just as we cannot definitively tell which effects the introduction of certain technologies will have on socio-material arrangements.

Specific to AI, an immediate effect that has been pointed out by many researchers is the role of automation in displacing jobs and concentrating economic and political power in the hands of those who control the material and algorithmic infrastructure that powers AI systems [13,36,37,57–59], while also negatively affecting the generation of public benefits [60]. While open source codebases do carry a promise of more ‘democratic’ access to technological development, the ability to develop, deploy and train new computational models that form the basis of many AI systems continues to be restricted to powerful actors with large-scale resources [5,7,13], and the assessment of risks associated with them remains the prerogative of selected elites which may not be affected by harmful outcomes to an equal extent. Such concentration of economic power can lead to inequalities becoming entrenched [38,55] and longer-term risks from how economic inequality interacts with society [26,56].

A second potential outcome is that automated systems contribute to maintaining social inequality via biases that are ‘learned’ or otherwise become part of them over history [7,9,10,13,41]. Artificial intelligence allows classificational systems to operate on unprecedented scales and in ways that are in principle or in practice opaque to inquiry [24,38]. As such, the epistemic practices associated with AI systems transcend the immediate context of their usage (creating an AI system for a particular purpose), potentially participating in complex feedback with the wider social context in which they operate [11]. Furthermore, due to inherent biases in underlying data, these algorithms can reproduce, reinforce and even resurrect inequalities, with little autonomy for the society to avoid or mitigate them [10,13,36].

If such classifications obtain a central function where their outputs become irreversible, they will continue to affect people’s lives long after the moment or purpose of classification has passed. For example, admittance to a university will affect a person throughout their life; thus, it represents a classificatory decision that is not easy to supersede at a later stage. One example with even wider-ranging effects of classificatory schemes is the usage of race classification in Apartheid South Africa as discussed by Bowker & Star [51]. When much of the access of a person—to places, to opportunities, to rights—depends on the classification of belonging to a particular group, this classification enacts particular social realities and can be exceedingly difficult to change. With AI systems, another layer can emerge where the opacity of the system combined with the segregation of expert and lay knowledge (often rendering the latter irrelevant) can make it especially difficult to contest outcomes that more easily become part of newly constructed social realities [6,37]. In this regard, the use of AI in the criminal justice system can be especially concerning as opportunities to contest decisions are limited [11,24]; similarly, use cases in urban planning can lead to such issues given the history of marginalization of disadvantaged communities in planning decisions [61,62].

Possible interactions with existing inequalities should be prominently present in risk assessments. These interactions should be integrated into a complex systems perspective to better account for possible emergent outcomes from a variety of technological, environmental and social forces that can act to increase or decrease inequality.

Multiplicative processes, in their nature, result in long-tail power-law distributions such as wealth inequalities, and such processes of wealth accumulation can, thus, result in significant inequalities in wealth and power unless counteracted explicitly [63–66]. This dynamic of concentration and decentralization occurs in other complex systems in nature [67]. Therefore, inequality is not only enacted in particular times and places through differentiation between certain people with the consequence of unequal access to resources, be those materials or in the form of social or cultural capital; in some cases, inequality becomes entrenched over time through the cumulative or compound effects of interactions between systems [68,69]. Research shows that social institutions play an important role in managing or avoiding such inequalities [63,70–72].

## 4. Artificial intelligence systems and political participation

A more specific type of inequality is inequality in how individuals can participate in politics and collective decision-making. Human societies, both historically and in modern times, vary immensely in their collective decision-making structures and the forms and degrees of political participation they allow for different segments of society [73]. While recent debates typically focus on formal institutions and political forms, in any society, it is important to consider: (i) the role of business elites in important decisions and any oversight by governments and the public, (ii) informal institutions that affect political and business decision-making, and (iii) legitimacy of institutions, regulations and interventions, either by governments or by public actors [74,75].

Considering the deployment of AI systems, interactions with collective decision-making processes will likely form complex feedbacks. Technologies deployed can drastically affect how formal or informal participatory processes work, by raising or lowering inequality in access to them, e.g. by granting disproportional influence to certain actors [26,38]. At the same time, collective decision-making processes will influence how new technologies are deployed [36,37,76–78]. Importantly, the deployment of technologies and policy is typically done by a limited segment of society: technology leaders, highly educated workers, businesspeople and politicians [23,30]. Such a segment is often labelled as ‘elites’ based on their disproportionate influence on public policy. However, the full effect of technology is realized only based on its interaction with the wider public and is influenced by individual attitudes and decisions [38,79,80], along with complex social interactions that significantly affect technology adoption [81,82]. Policy decisions (e.g. regulation of technology use) will need legitimacy from the wider public, while at the same time, emergent norms around the use of technologies will play an important role [6,83]. Considering such feedback is very important not only to better be able to assess risks from technology deployment but also to understand potential biases in such risk assessment processes themselves.

Historical research has found important links between governance structures, inequality and instability [73,84,85]. Arrangements that have favoured the rise of an elite segment that focuses too much on self-enrichment often led to inequality and instability rising together, potentially culminating in crisis periods with profound human costs both historically [86–88] and in modern contexts [55,89]. Concerns that the deployment of AI systems can lead to such arrangements have also been raised over the recent years [26,38]. In this regard, when thinking about the role of new technologies, we need to consider two issues that correspond to the two sides of the complex feedback loop outlined above: (i) how the governance of technologies could affect the governance of society, especially whether they can enhance or limit political participation, and (ii) how the *current* political and decision-making structures will affect the deployment and regulation of new technologies. Recent research has shown that depending on the context, AI systems could have positive or negative effects on political discourse and, thus, collective decision-making [20,50,90,91]; building on such understanding will also be important in evaluating possible regulation and interventions related to the direct use of AI in the political domain.

Effectively managing technological development in the future will need responses that appreciate the above feedback and capitalize on them to maintain and increase public participation in governance. Outcomes will depend not only on adopting any specific policy or regulation but also on the presence of *social resilience*, i.e. the ability of a society to make effective collective responses to changing circumstances [75,85,92–94]. In the case of global collective action problems, resilience means finding solutions that are not only effective but can also maintain function in the face of a changing environment, external shocks and internal disruptions. Resilience, thus, depends not only on increased and effective political participation but also on effective and constructive public discourse that is able to mitigate various sources of bias while maintaining necessary levels of cooperation. As technological shifts (such as the deployment of AI) always have a destabilizing potential [80], having sufficient social resilience can be crucial in successful adaptation.

## 5. A case study: UK school examinations during COVID

We illustrate some of the previously discussed issues by focusing on the deployment of an algorithmic solution in an attempt to prevent inequality. In spring 2020, final exams for GCSE, AS and A levels in the UK were cancelled due to the risks posed by the COVID-19 pandemic [95–100]. However, there was still a need to assign grades to students so as to allow them to progress with university entrance or job applications. The decision was taken to employ a predictive algorithm, and the UK’s Office of Qualifications and Examinations Registration (Ofqual) was tasked with developing it. At the time, the UK’s government believed that simply asking teachers to predict their students’ performance would undermine the confidence of sixth-form schools, universities and employers in the skills of their prospective students, as the initial results showed an uptake of good marks in comparison to previous years (13.9% would have received A* while previously, only 7.7% had obtained this grade), while further concerns were raised about the ability to compare grades between schools. Ofqual, thus, stepped in to protect the validity of the outcome of the examinations by introducing a process presumed to be more objective and, thus, fairer, relying on a statistical analysis of students’ performance in previous years. As Ofqual stated in their report:

Standardisation was not solely implemented to ensure that grades were not, overall, excessively high this year. The key purpose was to ensure fairness to students within the 2020 cohort. Without standardisation there was the potential for students to be unfairly advantaged or disadvantaged, depending on the school or college they attended and the approach they took. A key motivation for the design of the approach to standardisation that we took was to remove this potential inequality and, as far as possible, ensure that a grade represents the same standard, irrespective of the school or college they attended. [100, p 6]

This lengthy quote highlights a few important aspects. In a year that corresponded in few ways to normal processes, Ofqual’s aim was to ensure that the grades provided in one year are comparable to the grades provided in other years. At issue is effectively the notion of risk: while for Ofqual, the risk lay in students from certain schools or with particular teachers obtaining higher or lower marks than they might if they had taken the actual exam, other actors saw risks in a different aspect, as can be seen from the consultation report:

The risk arising from the award of a grade which is lower than that which a student would otherwise have achieved is greater than the risk arising from awarding a grade which is higher than would otherwise have been achieved. [101, p 14]

In this comment from the consultation report, risk is framed as risk toward the future developments of students at individual level. However, this comment is valid regardless of the means of assessment and could also be the reason why teachers tend toward awarding higher marks when predicting outcomes. Nevertheless, in the context of developing algorithms that could be drawn on again to predict outcomes, particular attention needs to be paid to potential perpetuating effects by assuming inequalities in grading will persist. The algorithm itself was developed based on historical data about student performance in previous years, which encompassed both their final grades as well as their previous performance, that was then formulated as a national average benchmark relation between prior assessments and actual grades. This model was validated based on the historical data of previous graduates and then used to predict the marks of 2020 graduates. As input to the prediction, both historical data about the performance of 2020 graduates and a ranking of students by assessment centre (i.e. school or similar) were used.

For the ranking, assessment centres were required to order students by their performance in each subject. Such ranking was performed across all classes. Already in the consultation report, concerns about this ranking, in particular, were raised because it did not factor in the relative distance between students (e.g. a high-performing student in an otherwise low-performing cohort), disregarded the difficulty of establishing a ranking per cohort instead of per class (e.g. multiple teachers might teach a cohort, and their assessments may be difficult to synthesize) and offered no possibility of placing multiple students at the same place in the ranking (thereby enacting increased differences between students) [101].

When Ofqual put its algorithm into practice, several issues emerged. First, we note that not all students were assessed algorithmically, since those subjects which had too few students or no prior data to warrant the application of statistical analysis were evaluated based solely on their teachers’ predictions [100]; this means that the equality that the algorithm was designed to ensure between years and schools was, from the very beginning, a mirage. Considering the actual predictions, there was a significant increase in grades A and above (despite the explicit goal of maintaining comparability among cohorts); however, approximately 40% of students did not receive the grades they reasonably expected and were instead downgraded by at least one mark [95,96]. While this could be expected given the variability of grades even among students with similar previous performance (thus, any model will be affected by stochastic variation), a more detailed analysis revealed that such outcomes are not randomly distributed: grade predictions clustered by postal code, with worse predictions affecting schools in disadvantaged areas disproportionately [98]. This means that prediction errors, thus, reflected existing inequalities in the educational system more than the performance of students. Students in private schools benefited most from the grade inflation, while students in disadvantaged postal areas were most negatively affected by it. Significantly, all this happened despite a previous assessment of the outcome to identify and mitigate bias [100,101].

This case demonstrates several important issues that arise when deploying large-scale algorithmic solutions or arguing about the risks associated with them. In order to better appreciate these, the AI system needs to be seen as sociotechnical, which is to say that it is not possible to reduce observation to the technical components alone; instead, the relevant level of observation is the AI system embedded in its context of use, including the people affecting and affected by the technical components. In the case of student ranking and university entrance exams, this means considering the educational system and its embeddedness in society as a whole to understand its full implications. The main issues to consider here are the following:

- There are significant inequalities in access to and quality of primary and secondary education. These will affect outcomes even if the grading system is perfectly ‘fair’. Any system used for university entrances will have only limited opportunities to compensate for these.
- The current university education emphasizes a pathway where students are immediately admitted after finishing secondary education. Many students who fail to enter a university right after high school will not be able to enter later.
- The ranking performed here has a very large impact on the life of students involved. University education is a main determinant in many outcomes later in life, in terms of economic achievement, health and wellbeing, but also in later opportunities for participation in public policy-making [55].
- This way, inequalities in educational attainment are a main contributor to maintaining social inequalities.
- The experience in the educational system has a possibility of affecting an individual’s perspective of their relation with the government and society. Individuals and groups that are systematically disadvantaged could decrease their trust in education and in the government in general.
- For those not affected negatively, it may be difficult to conceive of the consequences of negative outcomes for others. Differential experiences can, thus, translate to a polarization based on trust in important social institutions, such as the education system. Mitigating this would require translating differences of outcomes in ways that allow others to make sense of these differences in a meaningful way and allow for discussing and negotiating harms and long-term effects.

The above issues exist regardless of any algorithm performing a ranking function; however, the outcome of any algorithm will be affected by them. Thus, they should be considered more explicitly in algorithm design and risk assessments. In the case of Ofqual, while avoiding inequalities in grading was specifically among the goals, clustering of the grades suggests that existing socio-economic inequities, and inequalities in access to and quality of education were not given sufficient consideration when designing and testing the model. At the same time, even if such inequalities were possible to eliminate, since the algorithm necessarily works by predicting grades based on the ‘typical’ performance of students, it cannot be a perfect prediction of individual performance, and thus, predictions are expected to have errors. Even if the typical grades assigned by the algorithm are free of bias, biases could still persist in prediction errors. Since grades assigned at this stage have a long-lasting effect on the lives of the students, any systemic bias in such errors has the potential to exacerbate existing inequalities in the educational system and related socio-economic inequalities. An additional effect is in the perception of outcomes, not only among the students who might consider a grade unfair but also among the wider society, where outcomes could contribute to reinforcing stereotypes and biases. We note that in this case, a major contributing factor to prediction errors is the limited input data on which the algorithm operates. Including more data, such as information about the personal circumstances of students, could, in theory, help in reducing prediction errors; however, the use of such data would raise serious concerns about privacy while also allowing biases to persist in more subtle ways. The consideration of the personal circumstances of students is indeed something that teachers and educators take into account and that relates to empathy and compassion, elements that are lacking in algorithmic decision-making.

Such issues in the algorithm design also demonstrate structural problems with existing risk assessments: they neither take into account the wider context in which the assessed algorithms operate nor the conditions of their creation as artefacts of specialized knowledge. These issues could be mitigated by risk assessments that consider the social context and complex interactions [30], while also allowing wider participation from affected persons and groups to be able to better identify issues such as the ones outlined above [6].

In many cases, risk assessments are framed as a decision either on how to design an algorithm that minimizes a risk or on whether to deploy an algorithm or not. However, risks from algorithms (or AI) should also inform the decision-making process that contributes to creating the social context in which they operate. Specifically, in the case of the UK, the immediate context was whether to cancel the exams or hold them in person. We note that the latter choice also represented hard to characterize risks, as knowledge about COVID was rapidly accumulating after initial infection peaks in the spring of 2020 [102]. Assessment of risks of an algorithmic solution would then necessarily affect the decision process about cancelling the exams or even about considering further alternate options (e.g. holding exams online), which have their own potential risks dependent on the social context. Considering possible long-term outcomes, mitigation of risks can happen not only by designing better algorithms or by decisions about allowing or disallowing the use of certain data or algorithms. Interventions aimed at the social context in which algorithms operate, aimed either at a direct issue or at raising social resilience in general, can be equally or even more important.

## 6. Risk assessments through computational models and diverse competency groups

As mentioned above, as the effectiveness of risk assessments is conditioned on inequalities and a lack of participation of those affected by AI systems, we need methodological approaches that consider these factors. Yet this proposal of allowing for complexity in risk assessment and enabling democratic approaches in assessing risks leaves us with practical problems. Here, we outline two important directions that are central to risk assessments and that can form the basis of further collaborative, interdisciplinary experimentation.

### (a) Computational models and complex systems approach in risk assessment

Our above discussion highlights that AI systems need to be understood as an integrated part of our sociotechnical complex systems. Thus, a complex systems approach is necessary when handling adaptive dynamical systems with heterogeneous actors and interactions subject to uncertainties and emergence [27–29,33,103]. At the same time, we expect that AI methods will increasingly become part of complex systems research and the benefits and risks of such uses need to be carefully evaluated as well [4,42,62].

**Figure 2.**
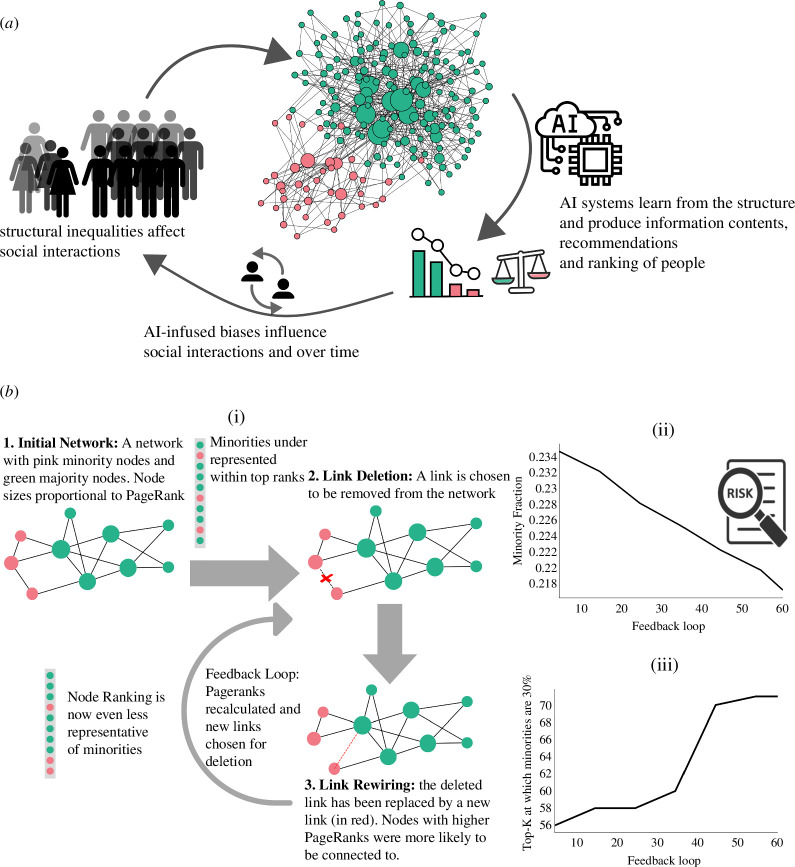
(*a*) An illustration of the complex system of AI-infused social networks. (*b*) A model of amplification of biases in networks due to the feedback between algorithm and human decision over time. Part (i), simulations are performed on a synthetic network of size 2000 with 30% nodes being minorities, and minority homophily and majority homophily are set to 0.7 each. In homophilic networks, minorities are less presented in the top ranks compared to their size, 30%. In each time step, five random links are removed from the network. They are then rewired, with higher-ranked nodes having a greater probability of being chosen as targets for the new connection. The ranking is recalculated, and this process of link rewiring is repeated over many feedback loops. The fraction of minorities in the upper ranks (e.g. the top 10%) reduces over time. The results are averaged over 10 independent experiments. In part (ii), we can observe the fraction of minorities in the top 10% goes from 23.4% down to 21.8% in 60 iterations. Part (iii) measures demographic parity, which demonstrates how far in the rank 30% of minorities are present as we expect, all else equal. At the beginning of the process, we achieve 30% representation of the minorities when we arrive at the top 56% of the nodes. At the end of the process, we need to include the top 71% to get this fair representation.

Let us examine a simple scenario of a social network with a numerical minority group and a majority group. The minority group can be taken to represent, for example, women or people of colour in computer science research networks. Societal biases such as homophily and in-group favouritism [104] shape the social networks in a specific way as shown in figure 2. Now let us consider a ranking algorithm that harvests this structural information to rank the most influential people in the field. The most prominent and widely used algorithm is PageRank [105]. Such ranking algorithms are commonly used in various flavours of professional social networking applications such as LinkedIn, Google Scholar and ResearchGate. We now consider how these rankings and biases set up self-reinforcing feedback loops over multiple time steps.

In line with previous research [106], we generate networks with 30% minorities and a moderate level of initial homophily (0.7). The ranking algorithm (PageRank) under-represents minorities in the top ranks. As can be seen in figure 2*b*, at the first time step, the structural position of minorities in the network influences how the algorithm ranks them, placing them lower than what we expect from their size. More importantly, minorities’ position in the ranking progressively declines as we let algorithmic visibility influence people’s choice in connecting to other highly visible (i.e. highly ranked) people. This is based on recent empirical evidence that ranking positions influence people’s decisions on what to click and which information to follow [107]. Concretely, in each iteration, five links in the network are ‘rewired’ to connect to other nodes in such a way that higher-ranked nodes are more likely to be the targets of the new links. This leads to a worsening of the under-representation of minorities (from 23.4% to 21.8% in the top 10% highly ranked nodes) over successive feedback loops of this process of rewiring and PageRank calculation. In other words, we observe a long-term decline in the ranking of minorities as a result of algorithmic visibility that we would not observe otherwise.

This computational model, by assessing the effect of AI-based algorithms on the minorities in the long-term, can, thus, be used to assess the risks and their evolution, especially if it is too risky to wait for the data to unfold. A similar approach has demonstrated the formation of more cohesive groups as a result of self-reinforcing feedback loops with link recommendation algorithms [108].

Similar computational models incorporating feedback were used in recent research to gain insights on cooperative solutions to important problems. Safarzynska & Smaldino present a model to explore links between inequality and cooperation globally [109], while Andrews *et al*. connect inequality, cooperation and sustainable resource use [72]. More directly related to the context of AI, Alalawi *et al*. focus on the issue of trust in a model where users make choices about using an AI system based on its perceived safety and benefits while also tackling the different perspectives of regulators and AI developers as groups with potentially divergent interests in an agent-based simulation [43]. Ensign *et al*. tackle the question of predictive policing and show that a feedback loop can lead to a runaway effect where policing efforts are allocated highly disproportionally, significantly affecting perceived crime rates as well [11]. On the contrary, Brinkmann *et al*. explicitly consider a malicious central actor (such as an oppressive government) employing an AI system and the possibility of adaptive responses from agents acting in a decentralized fashion to subvert it [110]. All of these studies demonstrate complex emergent outcomes and show how assessing them computationally can widen our perspective on possible social interactions that are relevant to evaluating risks of AI systems. Building on these approaches could allow integrating perspectives on AI, inequality and participation in collective decision-making that can form the basis of more informed risk assessments as well. This can help facilitate knowledge-building within a heterogeneous group of experts and laypeople.

### (b) Using competency groups to inform risk assessments

Finding effective ways of public participation in risk assessments can be a crucial component in ensuring that effects across wide segments of society are taken into account and that policy solutions will be successful in reaching their goals [103,111]. An important aspect is that public contestation and negotiation can help define and estimate risks in a way that acknowledges the epistemic diversity and represents the interests of affected persons. While involving a wider public in debates and decision-making about complex systems is not straightforward, many of the approaches used in complexity science are well amenable to wider public participation on different levels. For example, agent-based models, often combined with modern visualization techniques, offer tools where causal relations between complex interactions and outcomes can be easily grasped [112,113]. At the same time, such models offer the possibility of experimentation and incorporation of new insights that come from participatory exercises. Similarly, scenario planning exercises have been successfully used to explore possible uncertain futures in a collaborative setting [114]. Interventions that are explicitly based on empowering citizens and fostering public participation and engagement have been proposed in several domains, including healthcare, during the recent pandemic [115].

One promising way that can flexibly integrate such approaches to foster effective collective decision-making by public participation is the use of competency groups, as was demonstrated by Whatmore & Landström in their case study on flood mitigation measures [116]. In this example, a community in the UK frequently affected by flooding opposed the flood protection measures proposed by the Environment Agency. This opposition halted the deployment of flood protection measures but, of course, did not prevent the continued occurrence of floods, which led to the increasing entrenchment of the controversy. To slow down reasoning, prevent participants from jumping to foregone conclusions and allow for an opening of the controversy, a team of social and natural scientists engaged with people from the affected community to work together with them to understand, reframe and finally invent a new solution to the problem. Two central parts of this exercise were the reassessment of risks from the viewpoint of affected people and widening the scope of potential solutions by allowing direct public interaction with an extended set of underlying models. Eventually, the outcome was an innovative system strongly incorporating upstream storage and limited direct interventions, which subsequently became a popular intervention measure throughout the region.

We argue that a lesson can be learned from this case study for risk assessments in the context of AI systems: public participation can help make risk assessments and measures to mitigate against risks more effective. Through methods like competency groups, risk assessment models based on complex systems theory can be adapted in such a way as to allow affected communities to participate in decision-making about risks when it comes to AI systems [103]. In the risk assessment itself, the competency group could bring in different forms of knowing about the effects of AI systems in particular contexts. It could help level the divide of credentials separating laypeople experiencing harms caused by AI systems from experts speculating about potential risks—which, of course, is a very polarized example, as competency groups are likely to be much more nuanced. Importantly, this approach could be developed further to enable adversarial risk assessments, i.e. risk assessments conducted by mixed groups outside of the organization developing or deploying AI systems.

Competency groups involve the selection of a relatively small group of people from heterogeneous backgrounds, including laypersons and experts from different fields. They are not meant to comprise a representative sample of the population (of affected persons). Instead, the legitimacy of competency groups is derived from a process where members of the competency group explore options of risks that have meaning to them and assess their implications as well as potential mitigation measures. This requires methods of facilitation suitable for opening up discussion and can entail a lengthy process of exploration. These resources may not be available to assess every AI system, but competency groups or similar initiatives should at least be used for AI systems with a large scale and scope, such as the Ofqual assessment.

### (c) A potential outline for participatory risk modelling

Given the limitations of risk assessments discussed above, we would like to encourage researchers to experiment with new forms of risk assessments that address both the challenge of interacting factors and long-term developments as well as the tendency to exclude laypersons and affected persons from the determination and evaluation of risks. In our view, such approaches would need to address several questions:

- *Definitions of risks are context-dependent*: Although the documentation of harms and incidents involving AI systems will help identify common risks, it is unlikely that a full set of risk criteria can be developed against which to assess AI systems. Risks created or exacerbated by the use of AI systems will, therefore, depend on the context of use and can vary. In the Ofqual example, inequality is a risk that should have been evaluated, just as any algorithm with far-ranging societal impacts should be tested against its potential to increase or reinforce inequalities between different groups.
- *Composition of the group conducting risk assessments*: Risk assessments should be conducted by heterogeneous groups because heterogeneity is an appropriate means of approaching complex problems [117]. This entails a selection of participants who need not be representative but should have different kinds of stakes in the usage of an AI system. If the risk assessment for the Ofqual algorithm had been conducted by a group comprising students, parents and teachers, their definition of which risks matter, how these risks should be assessed and which measures should be taken to mitigate against them would likely have diverged from the risk assessment conducted by Ofqual.
- *Adequate consideration of complex interactions*: Both in the short and long term, technologies such as AI systems interact with society in ways that are difficult to predetermine. Additionally, AI systems interact with other technologies, be it hardware or energy used for processing, telecommunications used for transmissions or databases and/or datasets. These interactions are difficult to think through and communicate. To return to the Ofqual example, the effects of implementing a grading algorithm seem to have been challenging to understand and comment on in advance, showing the limits of a written consultation approach. By developing a simulation of the effects of the algorithm on different groups, as well as allowing the assessment group to explore the effects of tweaks and changes, it is possible that the risk assessment would have better identified negative outcomes for students and created appropriate countermeasures.

## 7. Conclusion and outlook

In this article, we outlined key issues with risk assessments as they are conducted today and propose a way forward that we believe can address weaknesses of existing risk assessment procedures. The core of our proposal is to allow more diverse forms of expertise into risk assessments, thereby opening up spaces for negotiation and contestation that involve affected people at the same level as experts. Models based on a complex systems approach can act as a tool to make long-term effects and complex interactions between risks more accessible to a competency group, offering a medium for deliberation that facilitates collective intelligence. At the same time, such collaborative exercises will be highly valuable for scientists studying complex social, economical or technological systems by directing their focus on issues with high relevance and importance and helping bridge top–down and bottom–up approaches to understanding and mitigating key social issues.

## Data Availability

Code for the simulation may be found at [118].
